# MAFLD in Vietnam: a neglected public health challenge requiring urgent policy action

**DOI:** 10.3389/fcdhc.2025.1687149

**Published:** 2025-12-08

**Authors:** Thong Duy Vo, Huong Tu Lam

**Affiliations:** 1 Department of Internal Medicine, School of Medicine, University of Medicine and Pharmacy at Ho Chi Minh City, Ho Chi Minh, Vietnam; 2 Department of Gastroenterology, University Medical Center Ho Chi Minh City, Ho Chi Minh, Vietnam

**Keywords:** fatty liver, non-alcoholic fatty liver disease, MAFLD, Vietnam, metabolic syndrome, epidemiology, public health, liver cirrhosis

## Abstract

Metabolic dysfunction-associated fatty liver disease (MAFLD) is rapidly emerging as a major public health challenge in Vietnam, driven by rising rates of obesity, type 2 diabetes, and lifestyle changes. Although it contributes significantly to morbidity, mortality, and economic burden, MAFLD remains under-recognized within national health strategies and is largely neglected in non-communicable disease (NCD) frameworks. This narrative review synthesizes epidemiological, clinical, and health systems data on MAFLD in Vietnam, drawing on studies published between 2015 and 2024 alongside international guidelines to evaluate their relevance in the local healthcare setting. The evidence indicates that MAFLD affects more than one-quarter of urban adults and is steadily increasing in rural populations. Barriers to effective management include limited diagnostic capacity, lack of standardized guidelines, insufficient awareness among clinicians, and underdeveloped multidisciplinary care models. Complications are exacerbated by the coexistence of hepatitis B virus infection and the growing burden of cardiovascular comorbidities. Together, these factors heighten disease severity and accelerate progression to cirrhosis and hepatocellular carcinoma. Given these challenges, urgent multisectoral action is needed. We propose a strategic national roadmap that incorporates MAFLD into NCD policy, expands primary care screening using simple non-invasive tools, and invests in health workforce training to improve early detection and risk stratification. Strengthening multidisciplinary collaboration and leveraging digital health technologies can enhance patient engagement and access to care. Finally, regional cooperation and Vietnam’s participation in international clinical trials are essential to accelerate innovation and policy response. In conclusion, MAFLD represents a neglected but pressing public health issue in Vietnam. Proactive and coordinated strategies are required to reduce its long-term health and socioeconomic impact and to position Vietnam as a regional leader in addressing metabolic liver disease.

## 1 Introduction

Metabolic dysfunction-associated fatty liver disease (MAFLD), formerly known as non-alcoholic fatty liver disease (NAFLD), has emerged as a major global health concern over the past decade. In 2020, a consortium of international experts proposed the adoption of the term MAFLD to reflect the underlying metabolic pathophysiology more accurately and to establish positive diagnostic criteria based on evidence of hepatic steatosis in the presence of metabolic dysfunction, regardless of alcohol intake or the presence of other chronic liver diseases (1, 2). This paradigm shift not only redefines disease classification but also enhances clinical utility by identifying patients with higher risks of metabolic dysfunction and liver fibrosis who might have been missed under the former NAFLD definition, thereby improving risk stratification and public health relevance. Despite these advances, MAFLD remains largely absent from Vietnam’s non-communicable disease (NCD) policy framework.

Globally, MAFLD is now recognized as the most prevalent cause of chronic liver disease, affecting approximately 25–30% of the general population (3, 4). Its prevalence is rising in parallel with increasing rates of obesity, type 2 diabetes mellitus (T2DM), and metabolic syndrome. Importantly, MAFLD is not only associated with progressive liver damage, including advanced fibrosis, cirrhosis, and HCC, but also confers increased risk of cardiovascular disease, chronic kidney disease, and extrahepatic malignancies (5). As of 2025, the term ‘MAFLD’ has not yet been officially adopted in Vietnam’s national medical or policy documents, although it is increasingly used in research and academic discussions.

In Southeast Asia, the burden of MAFLD is particularly alarming due to rapid urbanization, sedentary lifestyles, and dietary shifts. Vietnam, undergoing significant socioeconomic and nutritional transitions, has witnessed a sharp rise in the prevalence of metabolic disorders over the past two decades. However, despite its growing clinical relevance, MAFLD remains under-recognized and under-diagnosed in Vietnamese clinical practice. Furthermore, national data on disease burden, natural history, and outcomes remain limited, posing challenges for healthcare planning and policy development.

This narrative review aims to comprehensively examine the current understanding of MAFLD in Vietnam, with an emphasis on epidemiological patterns, diagnostic challenges, clinical implications, and management strategies. We also highlight key research gaps and propose directions for future investigation and public health action in the Vietnamese context.

## 2 Epidemiology of MAFLD in Vietnam

Epidemiological data on MAFLD in Vietnam remain limited but suggest a rising trend, consistent with global and regional patterns. In a multicenter study conducted in Ho Chi Minh City and Hanoi between 2015 and 2020, the prevalence of hepatic steatosis assessed by ultrasound ranged from 20% to 31% among general adult populations, with significantly higher rates observed among individuals with obesity, T2DM, and dyslipidemia (6, 7). Notably, a hospital-based cohort at the University Medical Center in Ho Chi Minh City reported that over 50% of patients with T2DM exhibited ultrasound-confirmed fatty liver, highlighting the close association between metabolic disease and hepatic involvement (8).

In rural populations, although the prevalence of obesity and T2DM remains lower than in urban areas, a steady increase in MAFLD has been observed due to changing dietary patterns, decreased physical activity, and increased consumption of processed foods. A 2021 study from the Mekong Delta region reported a MAFLD prevalence of 18.5% among adults, with metabolic syndrome present in over 40% of affected individuals (9).

The growing burden of MAFLD is further compounded by the demographic transition of an aging population in Vietnam. Elderly individuals with multiple metabolic comorbidities represent a particularly vulnerable group with a higher risk of liver fibrosis and cardiovascular complications (10). Moreover, there is an emerging concern regarding MAFLD in children and adolescents, particularly in urban settings, driven by increased sedentary behaviors and childhood obesity—though robust national data in this population remain scarce.

Compared to other countries in Asia, Vietnam’s MAFLD prevalence remains moderate but is on a sharp upward trajectory. For example, MAFLD prevalence in China and South Korea has exceeded 30% in several urban centers (6, 11), underscoring the need for proactive surveillance and targeted interventions in Vietnam to prevent a similar epidemiological shift. Because no national surveillance system currently exists, these estimates - mostly derived from urban or hospital-based studies—likely underestimate the true burden of MAFLD in Vietnam.

## 3 Diagnostic strategies and challenges

The diagnostic approach to MAFLD in Vietnam is evolving but faces several challenges related to awareness, access, and implementation of evidence-based tools. The transition from NAFLD to MAFLD criteria allows for a more inclusive and clinically relevant diagnosis, yet adoption in daily practice remains variable.

Current diagnostic algorithms rely on the detection of hepatic steatosis using imaging modalities such as abdominal ultrasonography, controlled attenuation parameter (CAP) via transient elastography (FibroScan), and computed tomography. Ultrasonography remains the most widely used first-line modality due to its affordability and accessibility, although its sensitivity diminishes in patients with low-grade steatosis and high BMI. According to regional data, ultrasonography demonstrates a sensitivity of 74–89% and specificity of 81–90% for detecting moderate-to-severe hepatic steatosis, although accuracy decreases in mild steatosis and obese individuals (12, 13). Compared with the controlled attenuation parameter (CAP) using transient elastography, ultrasound remains more feasible but less quantitative. As recommended in the 2025 APASL Clinical Practice Guidelines, combining FIB-4 or NAFLD Fibrosis Score with ultrasonography enhances diagnostic accuracy and risk stratification in resource-limited settings.

In Vietnam, MAFLD frequently coexists with hepatitis B virus (HBV) infection, affecting approximately 15% of patients in tertiary hospitals. Coexisting MAFLD and HBV accelerate fibrosis progression and increase HCC risk (14). In addition, hepatitis C virus (HCV) remains a relevant cofactor, particularly among injection drug users and older adults. Alcohol consumption is also a major contributor, with WHO data estimating a per-capita intake of 8.3 liters of pure alcohol per year. Current consensus recognizes the metabolic dysfunction-associated alcohol-related liver disease (MetALD) subtype, reflecting the overlap between metabolic and alcohol-induced liver injury in Vietnam.

**Table 1** summarizes the most common non-invasive diagnostic tools applied in Vietnam, along with their clinical characteristics. Despite the availability of these tools, several barriers impede widespread implementation. These include lack of standardized national guidelines, limited training in liver imaging interpretation among general practitioners, and absence of reimbursement for advanced diagnostics such as FibroScan in the public health system. Currently, non-invasive fibrosis scores such as FIB-4 and NAFLD Fibrosis Score are applied using international cutoffs. These have not yet been locally validated in Vietnamese populations, potentially leading to misclassification of fibrosis risk and underestimation of advanced disease burden.

**Table 1:** Non-invasive diagnostic tools for MAFLD in Vietnam.

Tool	Use in Vietnam	Advantages	Limitations
Ultrasonography	Widely available	Inexpensive, non-invasive	Low sensitivity in early steatosis
FibroScan (CAP)	Limited to tertiary care	Quantitative assessment, reproducible	Costly, limited access outside major cities
FIB-4 Index	Increasingly used	Simple, based on routine labs	Low specificity in young/elderly populations
NAFLD Fibrosis Score	Rarely used	Good NPV for advanced fibrosis	Complex calculation, underused in practice
ALT/AST levels	Routinely tested	Available nationwide	Poor correlation with histologic severity

CAP, Controlled Attenuation Parameter; NPV, Negative Predictive Value; BMI, Body Mass Index.

In addition, the overlap of MAFLD with other liver diseases (e.g., HBV infection, alcohol-related liver disease) complicates the diagnostic process. Unlike the NAFLD definition, MAFLD criteria permit co-existence with other etiologies, necessitating a more nuanced diagnostic approach. This is particularly relevant in Vietnam, where hepatitis B virus (HBV) infection remains endemic.

Overall, while non-invasive approaches hold promise for early detection and risk stratification, integrating these tools into primary care and raising clinician awareness are critical steps for improving MAFLD diagnosis nationwide.

## 4 Clinical and socioeconomic impact

MAFLD represents a significant clinical burden with far-reaching implications for both individual health and the national healthcare system in Vietnam. Although hepatic steatosis was once considered a benign condition, accumulating evidence now demonstrates that MAFLD is a progressive disease with potential to evolve into advanced liver fibrosis, cirrhosis, and HCC (13, 15). Moreover, MAFLD is intricately linked with extrahepatic complications—particularly cardiovascular disease (CVD), which remains the leading cause of death among these patients (16) (**Table 2**).

**Table 2:** Clinical and socioeconomic impact of MAFLD in Vietnam.

Impact area	Key observations
Disease progression	15–20% with significant fibrosis; up to 5% with cirrhosis at presentation
Extrahepatic complications	High burden of CVD, CKD, and T2DM
Coinfection with HBV	Accelerated fibrosis and higher HCC risk in dual pathology
Healthcare resource utilization	Increased imaging, hospitalizations, and pharmacologic interventions
Economic burden	Productivity loss; contributes to 10–15% of liver-related healthcare spending

In Vietnam, recent tertiary hospital data suggest that approximately 15–20% of patients with MAFLD have evidence of significant fibrosis (F2 or higher) using non-invasive scoring systems, and up to 5% may harbor cirrhosis at initial diagnosis (8, 12). These figures are concerning given the relatively young age of many affected individuals. A retrospective study at the University Medical Center in Ho Chi Minh City found that MAFLD patients with advanced fibrosis were more likely to be male, obese, and diabetic, and had significantly higher rates of incident cardiovascular events within a 3-year follow-up period (17).

The coexistence of MAFLD with hepatitis B virus (HBV) infection—endemic in Vietnam—poses another challenge. Studies show that patients with dual pathology (MAFLD + HBV) experience more rapid fibrosis progression and higher rates of HCC than those with HBV infection alone (14). This synergy exacerbates the clinical complexity and necessitates more aggressive monitoring protocols.

From a health economic perspective, MAFLD imposes substantial direct and indirect costs. Direct costs include increased utilization of outpatient services, diagnostic imaging, and hospital admissions due to liver-related or cardiometabolic complications. Indirect costs stem from productivity loss, absenteeism, and premature disability. Although precise national cost estimates are lacking, extrapolations from regional data suggest that MAFLD contributes to approximately 10–15% of total liver-related healthcare expenditures in urban tertiary hospitals (18, 19). Beyond direct medical costs, MAFLD imposes indirect economic burdens through reduced workforce productivity, absenteeism, and premature disability, contributing to measurable macroeconomic losses in Vietnam.

In a broader public health context, MAFLD is an impediment to sustainable development goals. Its growing prevalence among working-age adults risks eroding economic productivity, especially in a country like Vietnam with a large proportion of its population in the labor force. Despite this, MAFLD remains underrepresented in national health programs, which traditionally prioritize infectious diseases and maternal-child health.

Given its multidimensional impact, MAFLD must be addressed as a systemic disease requiring cross-sectoral strategies. Integrating MAFLD into existing non-communicable disease (NCD) control frameworks, improving data collection, and allocating resources for long-term liver care are imperative for minimizing its societal burden.

## 5 Management landscape in Vietnam

The management of MAFLD in Vietnam remains in its infancy, characterized by an absence of national guidelines, limited pharmacologic options, and underdeveloped infrastructure for multidisciplinary care. Current treatment approaches focus predominantly on lifestyle modification, aiming to achieve weight reduction, improve insulin sensitivity, and reduce hepatic fat accumulation. However, implementation in clinical practice is inconsistent, and patient adherence remains suboptimal.

### 5.1 Lifestyle modification

Lifestyle interventions, including dietary changes, increased physical activity, and behavioral therapy, are considered the cornerstone of MAFLD management (20). Clinical trials have demonstrated that a weight loss of ≥7–10% can result in resolution of steatohepatitis and regression of fibrosis (21). In Vietnam, low-cost lifestyle interventions such as calorie-restricted diets and community-based exercise programs have shown promise in pilot studies (22). However, the scalability of these initiatives is challenged by cultural dietary preferences, lack of trained dietitians, and limited patient education about metabolic liver disease.

### 5.2 Pharmacologic therapy

No specific pharmacotherapy for MAFLD has yet been approved by the Vietnamese Ministry of Health. Nonetheless, several off-label agents have been employed in selected patients with biopsy-proven nonalcoholic steatohepatitis (NASH) and significant fibrosis. These include pioglitazone, vitamin E, and statins—although their use is limited by safety concerns, physician unfamiliarity, and patient reluctance (23).

Recent advances in drug development, including GLP-1 receptor agonists (e.g., liraglutide, semaglutide), SGLT2 inhibitors, and investigational agents such as resmetirom and lanifibranor, offer considerable potential. Among these, GLP-1 RAs are increasingly prescribed in Vietnam for T2DM and obesity, with observational data suggesting improvements in hepatic steatosis (24). In Vietnam, GLP-1 receptor agonists such as liraglutide and semaglutide are approved for diabetes and obesity treatment but not specifically for liver indications. Moreover, they are not reimbursed under national health insurance, limiting their accessibility. However, high cost and lack of insurance reimbursement limit broader accessibility.

### 5.3 Healthcare delivery barriers

According to the Vietnam Ministry of Health’s National Strategy for the Prevention and Control of Non-Communicable Diseases (2015–2025) and the Vietnam Non-Communicable Disease Health Survey (VNDHS 2021), fewer than 20% of primary care facilities perform routine metabolic risk screening (25, 26). The WHO Country Report (2023) further highlights that fragmented service delivery, lack of trained personnel, and limited digital health capacity remain major obstacles to effective chronic liver disease management in Vietnam (27, 28).

Vietnam’s fragmented healthcare system poses significant challenges to the coordinated care of MAFLD patients. Key barriers include:

- *Lack of awareness:* Both primary care physicians and specialists often underrecognize MAFLD, particularly in non-obese patients or those with concurrent viral hepatitis.
- *Insufficient screening:* Routine screening for liver fibrosis using FIB-4 or transient elastography is uncommon in most primary and secondary care settings.
- *Limited* sp*ecialist access:* Hepatology services are largely concentrated in urban tertiary hospitals, leaving rural populations underserved.
- *Policy gaps:* MAFLD is not yet included in Vietnam’s national strategies for non-communicable diseases (NCDs), which hampers funding and prioritization.

### 5.4 Proposed strategic framework

To address these limitations, a tiered, resource-sensitive framework is proposed (**Table 3**). Integration of MAFLD care into existing diabetes and cardiovascular disease clinics could streamline services, improve detection, and reduce redundancy. Multidisciplinary collaboration involving hepatologists, endocrinologists, dietitians, and primary care physicians is essential.

**Table 3:** Proposed tiered framework for MAFLD management in Vietnam.

Level of care	Interventions
Primary Care	Risk assessment (BMI, waist circumference), FIB-4 score, lifestyle counseling
Secondary Care	Confirmatory diagnostics (ultrasound, CAP), initiation of pharmacotherapy
Tertiary Care	Advanced imaging, liver biopsy (if needed), access to clinical trials

## 6 Research and public health gaps

Despite the rising burden of MAFLD in Vietnam, the disease remains vastly under-researched and poorly integrated into national public health agendas. Several critical knowledge gaps impede effective disease control, ranging from epidemiological surveillance to therapeutic innovation and health systems research.

### 6.1 Lack of national epidemiological data

While isolated hospital-based and regional studies have reported local prevalence rates, there is currently no national registry or longitudinal cohort that captures the full scope of MAFLD across Vietnam. This absence of standardized surveillance data limits understanding of disease distribution, temporal trends, and at-risk populations. Furthermore, the reliance on ultrasonography rather than validated metabolic markers or elastographic assessment underestimates the true burden of advanced fibrosis and cirrhosis. The absence of national-level epidemiologic surveillance hinders accurate resource allocation, policy prioritization, and long-term planning, leaving MAFLD underrepresented in Vietnam’s health agenda.

### 6.2 Inadequate representation in public health policy

MAFLD is not currently included in Vietnam’s National Strategy for Prevention and Control of Non-Communicable Diseases (2015–2025), which focuses mainly on cardiovascular diseases, diabetes, cancer, and chronic respiratory conditions. This omission has important implications: it results in lack of targeted funding, limited programmatic interventions, and minimal public awareness campaigns. In contrast, other countries in the region—such as South Korea and Taiwan—have begun integrating fatty liver screening into metabolic disease programs, a model that could be adapted to Vietnam’s healthcare setting (29).

### 6.3 Limited clinical and translational research

The volume of peer-reviewed Vietnamese research on MAFLD remains modest. Existing studies are largely descriptive and cross-sectional in design, with few prospective investigations into disease progression, genetic susceptibility, or treatment outcomes. There is also a paucity of translational research exploring the unique metabolic and dietary risk profiles of Vietnamese populations. As an example, the role of traditional Vietnamese diets, rice-based carbohydrate load, and herbal medicine use on liver fat accumulation is not well characterized.

Furthermore, Vietnam has not yet participated in major international clinical trials for emerging MAFLD therapies. This restricts access to innovative treatments and delays regulatory awareness and approval. Institutional capacity building is needed to support ethical review boards, patient registries, and pharmacovigilance infrastructure to qualify for multinational trial participation.

### 6.4 Gaps in training and awareness

Another major barrier is the insufficient training in MAFLD among frontline healthcare workers. A 2023 national survey found that less than 30% of primary care physicians were familiar with the diagnostic criteria for MAFLD, and fewer than 10% routinely calculated FIB-4 or NFS scores (23). Postgraduate curricula in internal medicine, endocrinology, and family medicine also lack standardized modules on fatty liver disease.

Medical education reform is urgently needed, including:

- Incorporation of MAFLD into continuing medical education (CME) programs.
- Development of digital learning tools and clinical decision algorithms.
- Encouragement of interdisciplinary training among hepatologists, diabetologists, and nutritionists.

By addressing these gaps, Vietnam can develop a robust evidence base to inform policy, allocate resources more efficiently, and improve outcomes for patients living with MAFLD (**Table 4**).

**Table 4:** Recommendations for research and policy development.

Research area	Key recommendations
National Surveillance	Establish MAFLD registries; integrate liver health modules into existing NCD surveys
Clinical Trials and Innovation	Facilitate access to international drug trials; incentivize public-private research
Nutritional and Genetic Studies	Investigate ethnic-specific risk factors; assess impact of local dietary patterns
Health Systems and Policy	Include MAFLD in NCD strategies; allocate dedicated funding for screening programs
Education and Workforce Capacity	Implement CME; develop national training curriculum on metabolic liver disease

## 7 Future directions

The growing recognition of MAFLD as a critical public health challenge in Vietnam demands a strategic, evidence-based, and multisectoral response. Future efforts must transcend isolated clinical interventions and instead focus on building an integrated national framework that links prevention, early detection, clinical management, and policy implementation.

### 7.1 Integration of MAFLD into national health policy

The formal inclusion of MAFLD into Vietnam’s national non-communicable disease (NCD) agenda is imperative. This would facilitate dedicated funding for liver health, support the development of national guidelines, and drive multi-level interventions. Building upon existing infrastructure for diabetes and cardiovascular disease care can provide a cost-effective platform for MAFLD screening and management, particularly at the primary care level.

### 7.2 Development of national clinical practice guidelines

There is a pressing need to establish standardized, locally adapted clinical guidelines for the diagnosis, risk stratification, and treatment of MAFLD. These guidelines should align with global best practices (e.g., AASLD, EASL, APASL) while reflecting Vietnam’s resource constraints. An example would be the use of FIB-4 as an initial screening tool at the community level, followed by transient elastography referral only for high-risk patients.

### 7.3 Expansion of multidisciplinary care models

Given the multisystem nature of MAFLD, the future model of care should be team-based and patient-centered. Establishing dedicated “metabolic liver clinics” within tertiary hospitals, where hepatologists, endocrinologists, cardiologists, and dietitians collaborate, could greatly enhance care quality. In parallel, training primary care providers to deliver basic MAFLD counseling and risk assessment will expand coverage and accessibility.

### 7.4 Leveraging digital health and artificial intelligence

Digital tools can support MAFLD prevention and management by enabling risk prediction, clinical decision support, and remote monitoring. For example, mobile applications incorporating FIB-4 or NAFLD fibrosis score calculators, lifestyle tracking, and telehealth follow-ups may increase adherence and disease awareness. Vietnam’s rapid digital transformation provides an ideal environment to pilot such innovations.

### 7.5 Fostering research and regional collaboration

Vietnam should prioritize participation in international MAFLD consortia and clinical trials to accelerate access to novel diagnostics and therapeutics. Regional collaboration with ASEAN countries could enable shared epidemiologic databases, training modules, and research funding. Establishing a Vietnamese MAFLD Research Network would also encourage multicenter studies and elevate the national academic contribution.

### 7.6 Public engagement and awareness

Improving population-level health literacy is essential to change behavior and reduce stigma. National campaigns should highlight the link between MAFLD and lifestyle, particularly obesity and diabetes, and advocate for regular liver health checks. Involving community leaders, media, and schools will increase outreach effectiveness.

MAFLD represents a silent but rapidly advancing threat to Vietnam’s public health landscape. Its intersection with prevalent metabolic disorders, aging populations, and endemic viral hepatitis underscores the need for urgent and coordinated action. By investing in epidemiologic surveillance, strengthening health systems, and mobilizing public and professional engagement, Vietnam can position itself as a regional leader in the fight against MAFLD.

**Figure 1:**
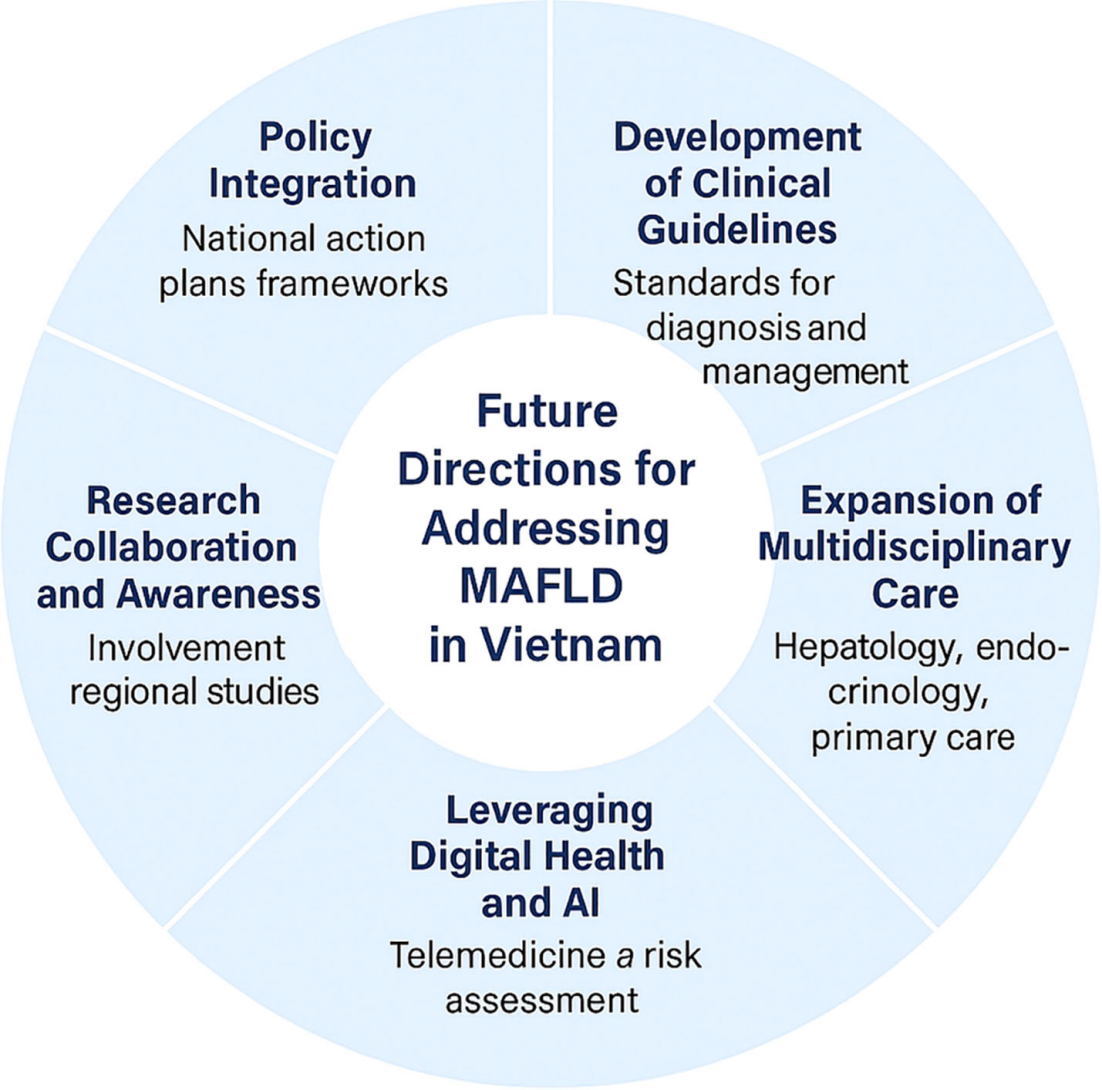
Strategic roadmap for MAFLD response in Vietnam.

Future success will depend not only on clinical excellence but on a collective commitment from policymakers, clinicians, researchers, and communities alike to prioritize liver health as a national imperative (**Figure 1**).

## 8 Conclusion

Metabolic dysfunction-associated fatty liver disease (MAFLD) is rapidly emerging as a dominant liver disease phenotype in Vietnam, driven by the accelerating prevalence of obesity, type 2 diabetes, and other metabolic syndromes in both urban and rural populations. The epidemiologic shift of MAFLD in Vietnam underscores a broader failure to recognize liver health as a public health priority. The disease imposes an escalating burden on healthcare systems, driven by insufficient screening, underdeveloped clinical guidelines, and the systemic neglect of early intervention programs.

The Vietnamese healthcare landscape now faces a pivotal inflection point. While MAFLD remains underrecognized in clinical practice and underrepresented in national NCD policies, the evidence is unequivocal: early identification and intervention can markedly reduce long-term complications and healthcare costs. However, isolated clinical efforts are unlikely to succeed without systemic changes. What is urgently needed is a paradigm shift—from reactive disease treatment to proactive, multidisciplinary, and population-based prevention strategies.

To this end, Vietnam must adopt a comprehensive national strategy that includes:

1. the formal recognition of MAFLD within national NCD frameworks;
2. the development and dissemination of locally adapted clinical practice guidelines;
3. the integration of MAFLD management into primary care;
4. the expansion of digital tools and AI to support early detection and patient engagement; and
5. a robust national research agenda with regional and global collaboration.

Ultimately, the success of MAFLD control efforts will depend on more than clinical expertise alone. It will require a unified commitment from policymakers, researchers, clinicians, and communities to elevate liver health as a priority on the national health agenda. Cross-sector collaboration between government agencies, academia, and civil society will be critical, offering Vietnam the opportunity to become a regional model for MAFLD prevention and policy development. With timely, evidence-informed, and multisectoral action, Vietnam can transform into a regional hub for MAFLD innovation and policy leadership.
